# Between epistemic injustice and therapeutic jurisprudence: Coronial processes involving families of autistic people, people with learning disabilities and/or mental ill health

**DOI:** 10.1111/1467-9566.13855

**Published:** 2024-11-18

**Authors:** Sara Ryan, Francesca Ribenfors, Magdalena Mikulak, Deborah Coles

**Affiliations:** ^1^ Department of Social Care and Social Work Manchester Metropolitan University Manchester UK; ^2^ Faculty of Health and Medicine Lancaster University Lancaster UK; ^3^ INQUEST London UK

**Keywords:** autism, coronial processes, disability, epistemic injustice, inquests, learning disability, therapeutic jurisprudence

## Abstract

Understanding how and why someone dies unexpectedly is key to bereaved family members. The coronial process in England investigates instances where the cause of death is unknown, violent or unnatural and/or occurred in state detention. Families are held to be at the centre of this process and the coroner's role has extended to concern about therapeutic jurisprudence, that is, how legal processes can minimise negative consequences for participants without jeopardising due process. Therapeutic jurisprudence involves unresolved tensions, however, and an epistemic power imbalance. Within the inquest, knowledge is produced, evaluated and contested, and epistemic privilege may be unevenly distributed. The inquest is also a process that, as we demonstrate, requires epistemic courage and resistance on the part of families. Families with relatives who are autistic, have learning disabilities and/or mental ill health can experience epistemic and structural injustice before an unexpected death which makes the distinctiveness of their experiences important to understand. Here, we report on a qualitative interview project which focused on how bereaved families experience the coronial process after their relative died in receipt of health and/or social care support.

## INTRODUCTION

When someone dies unexpectedly, it is important for family members and those close to them to create a narrative that helps them better understand what has happened. Central to this is learning how and why the person died and making sense of the loss in personal, practical or existential terms (Neimeyer et al., 2006). In England and Wales, the coronial process, headed by the Chief Coroner, investigates instances where the cause of death is unknown, violent or unnatural and/or occurred in state detention. The investigation which draws on medical and legal expertise is led by a local authority appointed coroner whose purpose is to understand the medical cause of death and reach a legal conclusion (Howarth, 2007). While the inquest is a legal process, it is also an epistemic process where knowledge is produced, evaluated, contested and wherein epistemic privilege may be unevenly distributed (Mason, 2011; Mikulak, 2021). It is also a process that, as we demonstrate, requires epistemic courage and resistance (Medina, 2013). Here, we report on a qualitative interview project which aimed to understand how family members of autistic people, people with learning disabilities and/or mental ill health, who died in receipt of health and/or social care support experience inquests.

Legislative change in the form of the Coroners and Justice Act 2009 (CJA 2009) has led to a representation of the bereaved family as being at the heart of inquests (Kirton‐Darling, 2022). The coroner's role furthermore extends to concerns about therapeutic jurisprudence, that is, how legal processes can be adapted to minimise negative consequences for participants without jeopardising due process (King, 2008). Therapeutic jurisprudence is a longstanding consideration with Waller (1994) suggesting coroners should listen to family views and offer genuine condolences. Therapeutic measures introduced include counselling services, appropriate information, sensitive communication and allowing photos in the hearing (Freckleton, 2016; King, 2008). There is, however, limited research exploring how these measures are experienced (Dartnell et al., 2019) and an argument that the complex relationship between bureaucratic and pastoral functions has yet to be even partially resolved (Tait & Carpenter, 2013). Concern has further been raised about the development of therapeutic framing which may lead coronial actors to interpret their role as providing closure for bereaved families (Kirton‐Darling, 2022).

Related to this is the distinction between technocratic approaches, in which explanations are presented in expert language by ‘neutral’ figures, and convivial (or community) approaches where families are understood as participants in the production of the explanation (Morgan, 2006). There is an inherent epistemic power imbalance however and competing perspectives within the current system mean the family perspective can be sharply contested (Kirton‐Darling, 2022).

Furthermore, while the inquest is held to be inquisitorial (Thornton, 2012), it can be experienced as an adversarial space (King, 2008); and, as a result, there is inconsistency in how coroners reach conclusions. Limited access to legal representation for families despite sustained calls for reform (Angiolini, 2017; INQUEST, 2020) can leave other legally represented parties, including state bodies, with the potential to impact on the process (Kirton‐Darling, 2022). These factors create a complicated picture in which coroners have a pastoral role in supporting bereaved families while facing ‘an army of lawyers’ representing state bodies (Walsh, 2015, p. 1). The coronial process has been described as an example of a medico‐legal forum in which social powerlessness and injustices are recognised and reconstituted through practices, and where calls for change can be hampered by financial considerations (Kirton‐Darling, 2022).

Our project focuses on the coronial experiences of families with a deceased relative who was autistic and/or had learning disabilities and mental ill health. These groups which include considerable overlap (Mutluer et al., 2022; Zeidan et al., 2022) have received little attention in coronial research despite increasing attention paid to deaths more widely. This is surprising given the enduring inequalities in mortality rates (Catala‐Lopez et al., 2022; White et al., 2023) and the tendency to misrepresent the causes of these deaths. Landes et al. (2020), for example, highlight how ‘learning disabilities’ were reported as the cause of deaths on nearly 50% of US death certificates. This is not only imprecise and harmful as it obscures deaths from preventable causes, it further undermines the reliability of mortality trends. The experiences of families of relatives who were autistic and/or had learning disabilities and mental ill health within the coronial process remain an important gap.

### Epistemic injustice

Alongside therapeutic jurisprudence, we draw on the concept of epistemic injustice, introduced by Fricker (2007, 2017) and further developed by others (Medina, 2013, 2017; Pohlhaus, 2012). Epistemic injustice refers to a ‘distinctive class of wrongs’ in which what people say is disbelieved or discounted because of who they are (testimonial injustice) or when a person's experiences are not understood, ‘when their meaning‐making capacities encounter unfair obstacles’ (hermeneutical injustice) (Medina, 2017, p. 48). These hermeneutical wrongs are central to ‘the very structure of our communicative practices’ and are ‘impersonal, widespread, and systematic’ (Medina, 2017, p. 49). Hermeneutical injustice occurs when collective interpretive resources include gaps which disadvantage people when it comes to understanding their experiences (Fricker, 2007). Hermeneutical injustice is structural and a product of social powerlessness (Fricker, 2017, p. 59), but the role of the epistemic agency of privileged groups and individuals is key in its production and reproduction (Fricker, 2017; Pohlhaus, 2012). Consequently, we should take interest in the epistemically marginalised narratives and accounts, as they carry epistemic privilege. As Fricker (2017, p. 59), quoting Harding (1991), argues, ‘Start thought from marginalised lives’. That is, methodologically, marginalised epistemic agents hold a distinct epistemic advantage through having less investment in maintaining the status quo than dominant groups. Ignorance is not neutral but is a substantial epistemic practice that demands examination (Alcoff, 2007; Mikulak, 2021; Pohlhaus, 2012). In this context of unjust epistemic terrain and ignorance, epistemic resistance and epistemic courage (Medina, 2013) are helpful tools that highlight the particular economy of credibility—to borrow Carel and Kidd's (2021) term—at work in the coronial processes.

Families of autistic people, people with learning disabilities and/or mental ill health can experience epistemic and structural injustice before their relative's death which makes the distinctiveness of their experiences important to understand, as has been argued about disabled people more widely (Mladenov & Dimitrova, 2022; Scully, 2018).

#### Families' experiences of the coronial process

Research focusing on families' experiences of coronial processes in largely UK and Australian literature relates to inquests involving missing people (Dartnell et al., 2019), suicide (Chapple et al., 2013; Spillane et al., 2019; Tait & Carpenter, 2013), Aboriginal deaths in custody (Razack, 2015) and work‐related deaths (Ngo et al., 2021).

Families describe wanting to find out what happened, why and to gain accountability (Ngo et al., 2021). A consistent theme is the lack of appropriate information which can generate a sense of pre‐inquest foreboding (Spillane et al., 2019). Research explores areas such as whether the right to life contained in Article 2 of the European Convention on Human Rights is engaged; communication; and the court room environment (Biddle, 2003; Dartnell et al., 2019). The time taken to hold the inquest which can stretch to months or years is differently viewed as delays can prolong stress and diminish evidence, while allowing families time to grieve or deal with death related administration (Dartnell et al., 2019; Spillane et al., 2019). The process is a contested epistemic arena. Families dissatisfied with the inquest felt the hearing scope was too narrow, key witnesses were not called, there were limitations in the expertise of the coroner, or the family was unable to challenge issues that contradicted their knowledge (Dartnell et al., 2019). Overt, subtle and mundane mechanisms within the process can silence families, underlining their lack of institutional power, in part illustrated by legal funding inequalities (Kirton‐Darling, 2022). These are evidenced by the sometimes insensitive and adversarial actions of legal representatives for those implicated in the death (King, 2008) and court formalities (Biddle, 2003; Snell & Tombs, 2011). Experiences could leave participants questioning the purpose of the inquest (Snell & Tombs, 2011).

There is also evidence that families value inquests and feel a sense of justice when certain conditions are met (Dartnell et al., 2019). These include being treated with empathy and respect; as an agent of knowledge; being able to question witnesses; the formal identification of systemic failings; and gaining answers (Biddle, 2003; Davis et al., 2002). Therapeutic jurisprudence involves and intersects with practices of epistemic justice and power sharing between coroners, relevant institutions, legal counsel and families.

For families of autistic people or people with learning disabilities and/or mental ill health, there may be additional contextual detail relating to experiences before the person died, which centre on experiences of structural injustice and oppression. A broad literature draws on epistemic injustice in relation to structural inequalities around mental health services (for example, Grim et al., 2019; Hultman & Hultman, 2023; Okoroji et al., 2023), and a consistent feature of parenting disabled children and/or children with mental ill health is the dissatisfaction families experience within health, education and social care systems (Matthews et al., 2021; McNeilly et al., 2017), particularly as they negotiate complex and contradictory relationships with professionals (Ryan & Quinlan, 2018). Petriwskyj et al. (2017) examine shifting power relations, uncertainty and the complexity inherent within these relationships, as parents access appropriate information and advocate for their children which can be time‐ and care‐intensive (McManus et al., 2011; Runswick‐Cole & Ryan, 2019; Ryan & Cole, 2009). Of relevance here is the suggested shift in focus to how life‐worlds assault those mired in them (Charmaz, 2010, p. 18) and how epistemic injustice offers tools to identify and analyse the mechanisms of these often‐invisible assaults (Mladenov & Dimitrova, 2022).

People with learning disabilities and autistic people die earlier than their non‐disabled peers due to inadequate health care, diagnostic overshadowing and medication side effects (Glover et al., 2017; Heslop et al., 2013; Hirvikoski et al., 2016). In effect, they experience structural injustice across their shortened lifetimes, which is insidious and effective because it is naturalised (Vasanthakumar, 2018).

Evidence suggests parents experience contributory injustice (Dotson, 2012) as professionals ignore their knowledge and understanding (Gill & Liamputtong, 2013; Lundeby & Tøssebro, 2008; Reardon et al., 2017). This is particularly important when people are not able to articulate verbally for themselves and parents or other family members speak on their behalf. Further, they are prevented from demonstrating epistemic agency (Scully, 2018), the constitutive parts of which include identity and dignity (Freeman & Stewart, 2019).

## METHODS

Qualitative interviews were used to explore the experiences of bereaved family members living within the UK, who attended an inquest into the death of a relative, diagnosed as autistic or with learning disabilities and/or mental ill health and in receipt of health and/or social care services at the time of their death. The study aimed to develop an understanding of these experiences and was undertaken in partnership with INQUEST, a UK charity that supports bereaved families.

Ethical approval was granted by Manchester Metropolitan University Research Ethics and Governance Committee (ETHOS Reference Number 24253). Ethical considerations involving unexpectedly bereaved people have been identified (Pearson, 2020) and FR who conducted the interviews paid particular attention to how participants appeared, offering breaks and the opportunity to stop if they found the interview upsetting. It is also important, however, to recognise that sharing stories can be empowering through enabling people who have been harmed to reassert their moral agency. As Vasanthakumar (2020, p. 5) suggests, ‘Testimony can thus protect victims' rational capacities and enhance their wellbeing’.

Recruitment was guided by purposive sampling (Palys, 2008) and information about the project was shared via project team networks, social media and INQUEST bulletins. Interested people were sent an information sheet and consent form and an interview time was organised by FR. Verbal consent was recorded before the interviews after participants had a further chance to ask questions.

Twenty participants (12 mothers, 4 fathers, 3 sisters and 1 daughter) provided insights into 18 inquests which took place in England or Wales between 2015 and 2022. All had some involvement with INQUEST from receiving information or advice, the provision of a case worker or joining the charity mailing list. We acknowledge participants may have acquired critical consciousness through this involvement making them sensitive to forms of injustice (Mladenov & Dimitrova, 2022).

The inquests lasted between 2 h and 15 days, 12 were Article 2 and 8 of these involved a jury. Of the relatives who died, 3 people had learning disabilities, 8 were autistic and 13 were described as having mental ill health. In addition, one relative was diagnosed with Pathological Demand Avoidance, one with Prader–Willi Syndrome and one was described as alcohol dependent. Their ages ranged between 13 and 67.

Data were generated via in‐depth interviews lasting an average of 70 min (with a range of 30–112 min) using Teams, Zoom and, in one case, phone, conducted between November 2021 and September 2022. Following Ryan (2018), the interviews began with an open‐ended question to encourage participants to freely narrate their experiences, and interruptions from the researcher were minimal. Further interview questions, developed from literature, INQUEST family listening days (https://www.inquest.org.uk/family‐listening‐days) and discussion among the research team, were only used when conversation faltered or towards the end of the interview to ensure key topics were covered.

Interviews were professionally transcribed and anonymised by replacing identifiable text with descriptive tags, and participants were offered the opportunity to review their transcripts.

A thematic analysis involved coding the data using the organisational support of Nvivo software where all transcripts, analysis documents and reflections were stored. Following Braun and Clarke (2014), codes were grouped into analytic categories and provisional themes identified. These were revised and refined in an iterative process that involved close reading and periodic discussion with the research team (Smith & McGannon, 2018), moving back and forth between the data set and coded extracts to develop a more conceptual analysis. This led to the identification of two main themes and sub themes:

1) ‘The “heart” of the coronial process’ with sub themes, ‘very necessary counsel’ and ‘the aftermath’.
2) ‘The inquest as a continuation of previous experiences’ with sub themes, ‘standing on ignorance’; ‘not being listened to and feeling blamed’; ‘undertaking labour’ and ‘the importance of peer support’.

## FINDINGS

### The ‘heart’ of the coronial process?

Our findings suggest the family is not typically at the ‘heart’ of the coronial process. This is unsurprising given competing perspectives of technocratic and participatory approaches and entangled invested interests of the state institution(s) involved. Relatedly, there were few examples of therapeutic jurisprudence, although the benefits of this approach, which included the coroner engaging with the substance rather than formality of family involvement, treating participants with care and respect, listening to their requests, and showing an interest in the person who had died, were clear.

One participant described giving evidence at their son's inquest as ‘better than they feared it would be’, attributing this to the coroner's approach, while a second described the process as healing.

I think we were very, very lucky. We had an excellent coroner. I know some people don't, but he was excellent. And he was very gentle, very kind, and he just kind of guided me through. […] And he started off by asking me to just kind of paint a picture of [son], and what he was like, which was nice, and to be able to give the jury that opportunity to know [son] really.

(015)

… we found it very healing and… prior to the inquest I hadn't slept through the night since she died, or a couple of weeks since before she died and the night after I slept right the way through and have done almost every night since. That sort of shows the power it had in terms of giving us answers and how important those answers were. Not just about why things went wrong but also what went wrong because we just didn't have a picture or a story in our mind. We just didn't know. So that was really important for us.

(002)

There was articulation of the epistemic advantage participants had which one participant explicitly connected to why families should be at the heart of the process:

The only thing that people want, when someone dies, is take responsibility for it, be open and honest, and make the changes. That's all we want. So that's why we should be at the heart of the process, because we're the ones that really want, I think… really want to see change. So, you know, we're not just like, an inconvenient thing that the coroner might have to talk to or not.

(001)

Following Spillane et al. (2019), we also found evidence of feeling apprehension and a sense of foreboding. Participants described being alone and out of their depth as they navigated unfamiliar processes at a time of unexpected bereavement, arriving in the coronial system as ‘amateurs’, surrounded by experienced professionals who understood the language, law, legal rights and rituals, such as standing up when the coroner enters the courtroom.

It was bizarre. Really bizarre, because we'd never been into court before. And you know, it's like, “be upstanding” and I was like, what?! You know. And it was just, it was just completely bizarre. Completely… and you know, didn't know what on earth was going on or anything.

(011)

Everything was so… everything was so… unknown. Of course, it was, everything was totally unknown about it. We knew nothing, we knew absolutely nothing.

(001)

This bewilderment and sense of strangeness could be compounded by a lack of information and sometimes inaccurate advice from the coroner's office, hospital trust or provider. For example, one participant was wrongly told by the NHS Trust that the inquest would not be an Article 2 inquest, and some participants were led to believe that the process would not be adversarial via information shared by the coroner's office.

Other participants recounted being told, or reading, they did not require legal representation despite coming to realise this was crucial to securing a satisfactory outcome as we go on to discuss below. As 004 said, ‘And all the advice on inquests says you don't need legal representation. It's a family friendly process’.

Procedural issues such as a lack of a named person to contact could contribute also to feelings of disconnection and isolation.

In the coroner's, it was awful. So […] the organisation was shocking, so we, all you were given was a generic email address and that was it. You were given no phone number and no personal email address and I was given a contact name but I'd never know, there was a lot going round […] so you'd send emails to this generic email address and put “for the attention of” and then you may or may not hear back and you'd have no idea whether they'd received it.

(002)

The powerlessness of family members could be reconstituted and further worsened by the autonomy of the coroner, the lack of a standardised approach, financial inequalities and absence of a complaints process (bar judicial review) when participants felt the process had not been fair or comprehensive. Examples of life‐world assaults included experiencing the coroner's approach as rushed, rude or dismissive.

…we all sort of think that, until this stage happens, we all sort of think that inquests are about how, finding out, how have people died? Well, I, you know, my experience is at the – and a lot of other people apparently – is that it seemed to be more of a wanting to get it done really quickly, with as little interference as possible from the people involved.

(014)

Furthermore, there was disagreement with the decision around the scope of the inquest or the requirement for an Article 2 hearing. Participants felt that the coroner should take into consideration failings in health and social care support that could go back months before the person died. For example, one participant was frustrated her daughter's inquest was not an Article 2 inquest and believed the coroner did not take into consideration the factors contributing to her death.

It just felt that, you know, had it been his daughter who died in those circumstances, he would have dug as we dug, he would have found out, and he would have asked those questions. And he wouldn't have entered “That's not relevant”. Because how can that not be relevant?

(017)

#### Very necessary counsel?

Similar structural and procedural challenges to those identified in existing research included inequalities around access to experienced legal representation which was typically perceived as integral to a ‘positive’ outcome. Some participants were able to fund legal representation which could be organised with the support of INQUEST, some pro bono support, for example, through a family friend and some had no legal representation. Support offered by legal counsel could include wider emotional support and dealing with media interest. Some participants reflected on how the inquest would have lacked a satisfactory level of scrutiny without this expertise.

You know, I had a lawyer, right? So, without that, like, what would I have? I'd have no information, misinformation, no legal support. No support, no knowledge. Like the whole system is set up in a way to disempower bereaved families, and to not to be able to make the changes.

(001)

This participant was concerned as it was only ‘by chance…by complete fluke’ a friend of a friend was an inquest lawyer who agreed to represent the family pro bono. Similarly, another participant (002) commented ‘You know I think if we hadn't had that legal representation, I think our inquest would have been a day at most’. They expressed concern for people without legal representation as they believed this contributed to having a 2‐week Article 2 inquest. These assertions were corroborated by participants who did not have legal representation. For example, one participant believed mistakes in evidence would have been spotted sooner which would have made the inquest ‘completely different’ (012).

Our analysis also identified barriers generated through a lack of understanding around how to work with a legal team, again speaking to the epistemic privilege of the counsel. In both examples below, the families had legal representation but experienced difficulties navigating their relationship with them and with the impact of their involvement:

…although they say that the coronial process has changed and it puts the family at the centre of the process, or that's what they allude, the problem is that once you appoint a solicitor or somebody to act on your behalf, you don't actually become the centre. Your centre becomes this third party…, who is there to help you navigate the system […] what we found is that we got cut out as soon as the lawyer become involved and number two, the lawyers were often advising us not to submit something we were interested in asking, because we didn't want to upset the coroner, we didn't want to burden the jury with things, too much information, so we did find that we lost control.

(005)

The other thing was, dealing with the legal team, having no experience at all of dealing with these guys, I mean, I was often asked by the barrister, who said, “Well, how would you – how are you going to direct me like this?” And […] it would have been really useful to have some guidance about how you can use the legal system […] It's like having a sort of computer without having a manual. You know. Like, how does this work?

(014)

There is a paradox here around the need for experienced legal counsel to facilitate accountability for families while the involvement of counsel (for those able to access them) may lead to further epistemic marginalisation.

#### High and dry: The aftermath

Following Dartnell et al. (2019), our analysis suggests the importance of considering the aftermath of the coronial process and impact on families. Participants described being ‘absolutely drained, just completely wiped out’ by the process and their involvement in it. One participant described struggling with their own mental health and believed her other children had ‘lost’ her through years of fighting for accountability. For this participant and the second participant below (012), the stress of the inquest resulted in ‘the grieving and the actual coming to terms with what's happened hasn't happened at all’:

I know I haven't, [processed the loss] and I can't even, can't even think about [daughter]. It's just too painful. I just had to put her things away in a drawer. And I can't. I just can't. So it's been – and I think the trauma of the inquest just makes that worse, doesn't it? Because it's – it takes so long, it takes so much energy, just dealing with the loss gets put back, and you just deal with the immediate.

(017)

Well, our lives are on hold. It's like the pause button has been pressed. And we lost, well, we'd lose him anyway because life is never gonna be the same without [son]. But we could have started to rebuild. We could have grieved the way we wanted to. Instead, I'm filled with anger. You know, and I don't cry. and I don't look at my son's picture and cry like I should. Because I'm so angry and because it doesn't allow you to accept the death. You can't accept it. Because you need the conclusion to be right. It needs to be the correct conclusion and you can't grieve because of that. I need the right answer.

(012)

The importance of recognising and acknowledging failings, receiving an apology and of demonstrable change occurring as an outcome was also clear. When these aspects do not happen, families can experience further harm and face continuing work in the form of seeking accountability. Coronial processes can therefore reinscribe and reinforce power imbalances and the continuing dehumanisation of the person who died (Goodley, 2020).

P2 And I suppose my naivety of originally an inquest, I know it's not to determine whose fault it is and that lot, but it's to get answers. That's your one time that you're going to get answers. And then if you're not given the answers that you're looking for, then you just walk out and you're just like…

P1 What was the point?

P2 I don't understand, I've just put myself through this.

(009)

And, you know, if something's got to be done, you do those jobs, basically, you know, I would say, like an emotional jellyfish, you know, just sort of, you know, you're doing those things to fill the time because you're not sleeping well, you know – all the stuff that goes through your mind, if I could have done this better or that better, you know, so you're not sleeping well. And there's loads of like formalities you have to deal with. So afterwards you're left high and dry, I suppose high and dry is the word.

(014)

In some cases, participants were left wanting a second inquest and some were pursuing a civil claim. One family, who did not have legal representation, believed a second inquest was ‘the right thing to do’ after failings were discovered by an independent investigation after the inquest, leading to the participant describing the inquest as ‘a sham’. We suggest these actions are examples of epistemic courage as bereaved families demonstrate their determination to generate change and accountability.

### The inquest as a continuation of previous experiences

Participants described experiences that echoed epistemic injustices they encountered before the death of their relative including a lack of understanding about learning disability, autism or mental health, feelings of not being listened to and blame and undertaking considerable unpaid labour. They also emphasised the importance of peer support.

#### Standing on ignorance

Our analysis suggests the coronial epistemic landscape can be dominated by ‘experts’ who exercise their epistemic privilege regardless of how knowledgeable they are. This led to inquests imbued with unchallenged practices of what could be described as ignorance by expert witnesses and coroners. Misunderstandings about the person and their support needs when they were alive were perpetuated as they seeped into evidence and potentially impacted the outcome. Participants described professional witnesses who had not met their relative, yet their evidence was given greater consideration (echoing the harms of testimonial injustice identified by Fricker (2007) and others), limiting participants' engagement and development as epistemic subjects (Dohmen, 2016).

I think, well I think the doctor's evidence was taken, you know, by the jury and been by the coroner, at absolute face value, he could have been saying – he could have been saying anything. And I believe that that would have been believed.

(014)

The knowledge of the coroner, by their seniority and official authority within the proceedings, could be a central feature of this ignorance which perpetuated what participants perceived as injustice and a lack of accountability, pointing to distinct hermeneutical harms. One participant directly linked a lack of challenge by the coroner about the actions of health professionals to the coroner's lack of understanding about autism:

And that was quite problematic because there seemed to be this real lack of understanding all the way through and to the expert independent witness that the coroner called, […] didn't know autism at all, and that quite shocked us because we weren't given a choice in who the expert independent witness was. Basically, the coroner himself chose, which is fine, but then he had, I asked him do you know about autism? He said “Oh yes, I know, we have been trained on autism and learning disabilities”, but it was very clear from his evidence that he had no understanding.

(002)

This brings us back to the specific forms of epistemic injustice disabled people experience (Scully, 2018). In this instance, the lack of specialised knowledge developed through the distinct experience of being autistic or being the family member of an autistic person fails to enter collective epistemic resources and remains misunderstood. We suggest this hermeneutic injustice may be implicated in the premature deaths of people with learning disabilities, autistic people and people with mental ill health and the engagement with these deaths in the coronial process.

#### Not being listened to and feeling blamed

Integral to this misunderstanding was a perceived devaluation of the relative within the coronial system as they had been in life and associated assumptions which again could influence the inquest outcome. This testimonial injustice and the dominance of technocratic accountability that carries epistemic privilege meant the person who died could be overlooked and the bereaved family shut out as in the examples below where participants believed the coroner and witnesses did not give their relatives the care or consideration they deserved:

I think the coroner just was really arrogant. Yeah, didn't understand the condition, didn't… and just couldn't be arsed.

(007)

P2 I think he thought, “This isn't worth considering because she's just some… girl…”

P1 Girl with a mental health problem…

P2 ‐ with a mental health problem, who's walked into the wrong place at the wrong time.

(004)

Participants expressed the importance of speaking at the hearing and showing photos of their relatives to assert their position as courageous epistemic agents with in‐depth knowledge of their loved one. This introduced additional labour which, if refused, could lead to further injustice.

I wanted to read the pen portrait, so I did that. I don't really like speaking in public, but I did actually want to do that. And I did do it. […]

I And what was the purpose of the pen portrait, then? To give a sense of who [daughter] was?

Yes. And also because I think I was sick of everybody else speaking for her, because they didn't know who she was.

(017)

While one participant said a video of her daughter, photos and examples of her artwork were shown, others experienced resistance from the NHS Trust or local authority which led to the coroner limiting or refusing photographs at the hearing. One participant described how the coroner allowed the legal teams representing the family and the hospital trust to debate the issue with the trust arguing the photos would unduly influence the jury:

We were not allowed to show our daughter's picture. We were allowed to show her on the day that we spoke, up until lunchtime, and then we were to take down her photo and never show it again, because that could adversely affect the jury's opinion of the case and make it too emotional when in effect it's not supposed to be an emotive thing. It's supposed to be about figuring out the how.

(005)

Participants were compelling witnesses to the life and death of their relative, yet this knowledge, courage and epistemic advantage were often discounted leading to tensions between the family, coroners and expert witnesses. This form of ‘smothering’ witness testimony by epistemic privilege being given to professional ‘objective’ knowledge is well documented within health‐care settings (Kidd & Carel, 2017; Mladenov & Dimitrova, 2022).

Participants also described feeling humiliated, blamed or that the death was somehow seen as the fault of the person who died. In these instances, epistemic injustice verged on epistemic violence. One participant felt the legal team representing the local authority was concerned with protecting their client and sought to place blame on their teenage daughter:

It was proper like a proper trial, wasn't it? I suppose, is… that's what I would… it wasn't a finding facts mission, it was a point of, right, let's put it all on [daughter], rather than fact‐find, let's put it all on [daughter], it's all her fault, so we're not at fault.

(009)

Another participant similarly believed the health trust was keen to attribute blame to their son who had died. During the inquest they struggled, not being able to respond to what they felt were inaccuracies in the evidence:

…they were saying things that weren't true, and I was getting quite upset about that. But I couldn't say outright, you know […] I found that was really hard, not being able to actually say.

(015)

#### Extensive unpaid work and cost to families

Several participants described the administrative labour they undertook which included learning about coronial processes and conducting investigatory work around what had happened. This was presented as necessary and traumatic work in part because ‘nobody was listening’. For example, one participant described the stress arising from the inquest and the work involved:

It was all very… you know, it was all quite stressful. But… [Sighs] what I can say, I felt… I mean… erm… [Pause] Well it was just something I felt I had to do, I had to go through. And it was trying to remember everything, because… and you know, providing information to the lawyer and the barrister, because they were quite, naturally they were relying on me to give them information about what had happened, and so‐on. […] I just found myself waking in the middle of the night, remembering things, “oh yes, I must remember to say that”.

(010)

Another participant, who used money from a bank loan and remortgaged her house to fund her legal team, was concerned about how much she could afford and therefore took on the work to limit costs:

I mean, it wasn't in the agreement to do it, but I went through every single witness statement, every ambulance, you know, the ambulance reports. Post‐mortem reports. I went through everything with a fine toothcomb. Because I was worried about the finances, and I wanted to be all over it, in case they hit a point where actually, we couldn't have a legal team anymore. And I just had to do it on my own. And that was really traumatic.

(007)

This labour was often a continuation of the work participants had undertaken across their relative's life and their meticulous records could contribute to evidence presented at the hearing. For example, one participant kept an archive of their brother's care when he was alive including details of meetings, phone conversations and safeguarding reports. Although it required time and effort to make this information legible for others, she believed it was useful:

I had a lot of records which needed to be transferred to documents and so on […] But I was able to provide a lot of information, which they were were able to corroborate. They were able to cross reference. So they had a lot. And a lot of evidence that was factual. Which helped.

(003)

Another participant felt her paperwork trail, which included documented requests for help and concerns raised about the risks to her son's life, helped secure the Article 2 inquest:

Straightaway [the coroner] said this is without question an Article 2 Inquest, I've seen enough. So it all ties back in my view… well to that paperwork trail that I had. And maybe others don't have that paperwork trail.

(006)

#### The importance of peer support

The final example in this section demonstrates how participants resisted contributory injustice by seeking recognition for, and sharing, parental knowledge and understanding with other families. The importance of peer support in this resistance is well documented (Fricker, 2007; Mladenov & Dimitrova, 2022).

Even afterwards, see, I went to a couple of the meetings afterwards where you meet other people who are in not dissimilar situations. They were, they were like the ultimate form of therapy. It was incredible. Being in a room with people have been the loved ones bereaved, there is a sort of, as soon as you get in that room, there's a sort of, because it's a feeling, it's a shock, you know, it's like, and it's, it's a shock, which you can't actually describe to anyone but someone who's been through it just being close to that person. I mean, it's a very strange feeling but – and there's a sort of, you know, it is a feeling of grief and all that.

(014)

There's something about meeting people that get it. Although the circumstances aren't quite the same I found that people that haven't – who are not living this, have kind of been there. I mean they're really kind and empathetic, but there's something about actually speaking to people that are having to go through the same thing that I found quite helpful.

(017)

This collectivity is important because while epistemic courage is an individual virtue, its importance lies in being echoed by others (Medina, 2013, p. 229).

## DISCUSSION

Our findings add support to what is known about families' experiences of the coronial system, particularly inequalities around access to legal representation and the therapeutic indifference families can experience from a technocratic process. We introduce the experiences of families where the relative is autistic or has learning disabilities and/or mental ill health, which generate further considerations around epistemic injustice, courage and ignorance.

For families of disabled or autistic relatives, the inquest can become a space of further dehumanisation, a new platform for experiencing structural injustice, hermeneutic marginalisation and blame. Our analysis suggests accountability and change are key drivers for bereaved families; however, dismissing or discrediting the person who died or family members is an accepted strand of coronial practice. This suggests epistemic injustice can be a pre‐requisite within the current coronial system creating obstacles to operationalising therapeutic jurisprudence. It is difficult to see how the competing relations of technocratic and participatory approaches and associated inherent epistemic power imbalances can be fully resolved within a system which reproduces and reinforces these imbalances.

Indeed, following Mladenov and Dimitrova (2022), we view the inquest as a site of painful assaults on the experiences, bodies and identities of the person who died and their family members. Given stark differences in mortality rates for people with learning disabilities, mental health and/or autistic people and consistent accounts of unsatisfactory coronial experiences in our data, we agree with Razack (2015) that a sense of inevitability can be used to cover wider structures of injustices.

In her work on epistemic injustice, Fricker (2017, p. 59) outlines how a slippery slope ‘to bad faith and self‐interested or plain lazy denial is an ever‐present factor’ where what is being said is potentially challenging to the person being told *or* sits outside of their epistemic comfort zone. Our analysis suggests ignorance as epistemic practice should also be considered as coroners and other authoritative actors, such as expert witnesses, demonstrate accepted and unchallenged layers of understanding and paucity of knowledge which can be wilful. Ignorance can be closely related to and enable epistemic violence and the silencing of marginalised groups (Mikulak, 2021). The implication of this ignorance is that the state of epistemic knowledge about the deaths of people with learning disabilities, autistic people and people with mental ill health is compromised. While we agree with Scully (2018, p. 112) who argues, ‘the wider community is harmed if its epistemic resources could be richer and more accurate than they actually are’, the implications of this impoverished understanding for this group are severe. Understandings are unduly influenced by the hermeneutically powerful (Fricker, 2007, p. 155) rather than the subject group who may be able to readily articulate their experiences (Dotson, 2012).

Participants demonstrated a special kind of epistemic courage particular to marginalised groups (Medina, 2013) and, in coming together with other families, they are forming a community of epistemic resistance (Mladenov & Dimitrova, 2022), drawing on experiences of epistemic injustice that can often be traced across the lifetime of their relative.

Therapeutic innovations and interventions, such as the inclusion of photos, intersect with epistemic resistance but are variable in practice. Participants with positive experiences described the impact of small examples of therapeutic jurisprudence: the coroner asking to see photos of their relative at the end of the inquest, having a named contact at the coroner's office and being treated with respect. We suggest the lack of implementation of these often simple to enact examples underlines the enduring social powerlessness of families in this context described by Kirton‐Darling (2022) and the impoverishment of collective epistemic resources. Participants described feeling disrespected and even humiliated by coronial processes, and the importance of showing photographs may be part of reasserting their moral agency and that of their relative by proxy. Experiencing disrespect, in part through ignorance of learning disabilities, autism or mental health, effectively discounts any approach of therapeutic jurisprudence and compounds epistemic injustice experienced by the families.

Entangled within this powerlessness is the considerable administrative, investigatory and emotional labour families undertake to try to ensure their relative is visible and due processes are followed. This labour can cause further harm which can be magnified as it is undertaken from an underprivileged epistemic position underpinned by grief. There is a poignancy here that meticulous record keeping undertaken during a person's life can become key evidence in the hearing into the context of their death.

Whilst legal representation was seen as a key factor in gaining accountability, questions were raised about obstacles counsel could create and how families may not know how to engage with lawyers. These issues need further teasing out beyond broader arguments around inequalities of access to legal representation. It is also important that the process is not viewed as a finite event as the aftermath can be distressing for the family and unsatisfactory outcomes may lead to ongoing labour.

In terms of improvements, our analysis supports adjustments around better information, communication and listening to families. In addition, our analysis highlights the importance of increasing knowledge and understanding around disability and mental health issues, including legislation such as the Mental Capacity Act, and involving expert witnesses who understand these areas. In effect, this would mean a lessening of ignorance through the recalibration and/or creation of ‘new epistemic resources for knowing the (dominant and marginalised) world more adequately’ (Pohlhaus, 2012, p. 720). The coronial service plays an important public role with the preventative potential of ensuring those responsible for unexpected deaths are properly held to account, to ensure learning, improvements with active engagement and the respect of families.

## CONCLUSION

The inquest process is inherently unjust and can become a site in which autistic people and people with learning disabilities and/or mental health are further dehumanised, generating further hermeneutic and material injustices for families who have typically advocated for their relative in a society in which their value is discounted. Families are forced to continue to undertake considerable administrative and emotional labour to try to gain accountability and ensure the humanness of their relative is acknowledged. Ignorance of autism, disability and mental health can generate further harm and unsatisfactory conclusions. Just as existing systems and services often did not meet the needs of the person when they were alive, the inquest process could fail them in death as questions around what led to their death remained unanswered. The demonstration of epistemic courage and resistance by bereaved families in the face of epistemic injustice of the process is a clear indication of failings within the current coronial process which could be ameliorated, following Dartnell et al. (2019), by being human, compassionate, respectful and informed. For this to happen. epistemic injustices and violence need to be identified, challenged and addressed by the Chief Coroner of England and Wales, individual coroners and the legal professionals involved in these processes.

## AUTHOR CONTRIBUTIONS

**Sara Ryan**: Conceptualisation (lead); formal analysis (equal); funding acquisition (supporting); methodology (equal); writing—original draft (lead). **Francesca Ribenfors**: Data curation (lead); formal analysis (equal); project administration (equal); writing—review & editing (supporting). **Magdalena Mikulak**: Formal analysis (equal); writing—review & editing (supporting). **Deborah Coles**: Conceptualisation (equal); funding acquisition (lead); writing—review & editing (supporting).

## CONFLICT OF INTEREST STATEMENT

The authors declare no conflicts of interest.

## ETHICS STATEMENT

Ethical approval was granted by Manchester Metropolitan University Research Ethics and Governance Committee (ETHOS Reference Number 24253).

## Data Availability

All data requests should be submitted to the corresponding author for consideration. Access to all anonymised data may be granted following review.
